# Rods contribute to the light-induced phase shift of the retinal clock in mammals

**DOI:** 10.1371/journal.pbio.2006211

**Published:** 2019-03-01

**Authors:** Hugo Calligaro, Christine Coutanson, Raymond P. Najjar, Nadia Mazzaro, Howard M. Cooper, Nasser Haddjeri, Marie-Paule Felder-Schmittbuhl, Ouria Dkhissi-Benyahya

**Affiliations:** 1 Univ Lyon, Université Claude Bernard Lyon 1, Inserm, Stem Cell and Brain Research Institute, Bron, France; 2 Visual Neurosciences Research Group, Singapore Eye Research Institute, Singapore; 3 Ophthalmology and Visual Sciences Program, Duke-NUS Medical School, Singapore; 4 CNRS UPR3212, Institut des Neurosciences Cellulaires et Intégratives, Université de Strasbourg, Strasbourg, France; Washington University in St. Louis, United States of America

## Abstract

While rods, cones, and intrinsically photosensitive melanopsin-containing ganglion cells (ipRGCs) all drive light entrainment of the master circadian pacemaker of the suprachiasmatic nucleus, recent studies have proposed that entrainment of the mouse retinal clock is exclusively mediated by a UV-sensitive photopigment, neuropsin (OPN5). Here, we report that the retinal circadian clock can be phase shifted by short duration and relatively low-irradiance monochromatic light in the visible part of the spectrum, up to 520 nm. Phase shifts exhibit a classical photon dose-response curve. Comparing the response of mouse models that specifically lack middle-wavelength (MW) cones, melanopsin, and/or rods, we found that only the absence of rods prevented light-induced phase shifts of the retinal clock, whereas light-induced phase shifts of locomotor activity are normal. In a “rod-only” mouse model, phase shifting response of the retinal clock to light is conserved. At shorter UV wavelengths, our results also reveal additional recruitment of short-wavelength (SW) cones and/or OPN5. These findings suggest a primary role of rod photoreceptors in the light response of the retinal clock in mammals.

## Author summary

The mammalian retina contains a circadian clock that plays a crucial role in adapting retinal physiology and visual function to light/dark changes. In addition, the retina coordinates rhythmic behavior and physiology by providing visual input to the master hypothalamic clock in the suprachiasmatic nucleus through a network of retinal photoreceptor cells involving rods, cones, and intrinsically photosensitive melanopsin-containing ganglion cells (ipRGCs). In contrast, recent studies argue that none of these photoreceptors are involved in light responses of the retinal clock and propose that photoresponses are exclusively mediated by the UV-sensitive photopigment neuropsin (OPN5). Our study demonstrates that rods are required to phase shift the retinal clock, while melanopsin and middle-wavelength (MW) cones influence the intrinsic period of the clock.

## Introduction

The mammalian retina contains an endogenous timekeeping system that ensures the fine tuning of its physiology to daily changes in light intensity [1]. The retinal clock controls the timing of a broad range of essential physiological and metabolic functions (for review, see [2]), including melatonin release [1,3], dopamine synthesis [4], photoreceptor disk shedding and phagocytosis [5–8], expression of immediate early genes and visual photopigments [9,10], electrical coupling between photoreceptors [11–13], the electroretinogram b-wave amplitude [14], circadian clock gene expression [15,16], and visual processing [14,17]. The retina also plays a key role in photic entrainment of the central clock located in the suprachiasmatic nucleus (SCN). This response is mediated through intrinsically photosensitive melanopsin-containing retinal ganglion cells (ipRGCs) that also receive inputs from rods and cones [18–22].

Mammalian retinas retain in vitro their ability to be entrained or phase shifted by light [1,3,23–25]. However, the response properties of the retinal clock to light and the involvement of different photoreceptors is still subject to debate. The landmark study by Ruan and colleagues demonstrated that the retinal clock is phase shifted by broadband white light [23]. In their model, ipRGCs and/or middle-wavelength (MW) cones were proposed to mediate light-induced phase shifts through synaptic contacts conveying excitatory influences to dopaminergic amacrine cells [17,26–31]. Dopamine is well known to play a central role in the regulation of light-induced responses of the retinal clock [23,31–35].

In contrast, it was recently proposed that light entrainment of the retinal clock is mediated uniquely through neuropsin (OPN5), a UV-sensitive opsin [25]. OPN5 is a bistable photopigment expressed in the eye [36,37] and in cells located in the inner and ganglion cell layers of several species [25,37–39] as well as in other tissues such as testis, ear, skin, pineal gland, etc. [24,25,36,37,40,41]. Retina of mice lacking rods, cones, and melanopsin (*rd1/rd1;Opn4*^−/−^) were reported to exhibit PER2::Luc retinal rhythms that could be entrained by a light/dark cycle [24], whereas OPN5 knockout mice (*Opn5*^−/−^) failed to entrain [25]. However, the relatively long-duration and high-irradiance light exposures required to obtain a response at 417 nm do not rule out activation of rods, MW cones, and/or ipRGCs based on their spectral sensitivities [42]. Furthermore, in mice lacking the essential components of phototransduction signaling pathways present in rods, cones, and ipRGCs, UV light stimulation fails to drive any electrophysiological responses or significant FOS induction [43].

Together, these findings outline two nonexclusive hypotheses of light entrainment of the retinal clock: light responses are driven by the UV light–sensitive OPN5/short-wavelength (SW) opsin and/or by classical photoreceptors in the visible region of the spectrum. To determine the roles of different photoreceptors responsible in phase-shifting responses, we first established the dose-response properties for light-induced phase shifts of PERIOD2::Luciferase (PER2::Luc) retinal explants and showed that the retinal clock can be phase shifted by short-duration, low-irradiance light at 465 nm. We also find that PER2::Luc rhythm can be phase shifted by monochromatic light pulses in the visible part of the spectrum, up to 520 nm. The involvement of different photoreceptors was determined by quantifying the phase-shift responses in retinal explants from mice lacking either melanopsin, MW cones, and/or rods (respectively, *Opn4*^−/−^::*Per2*^*Luc*^, *TRβ*^−/−^::*Per2*^*Luc*^, *Nrl*^−/−^::*Per2*^*Luc*^, *Opn4*^−/−^::*TRβ*^−/−^::*Per2*^*Luc*^) as well as *Opn4*^−/−^::*rd/rd*::*Per2*^*Luc*^, which lack all these photoreceptors. Our findings reveal that the absence of rods but not of melanopsin or MW cones prevents a light-induced phase shift at 520 nm and further suggest an additional contribution of SW cones and/or OPN5 at shorter UV wavelengths.

## Results

### Temporal and irradiance responses for light-induced phase shifts of the retinal clock

Although bioluminescence monitoring of PER2::Luc retinal explants has been used in several studies [23–25,44,45], a standardized procedure to determine the circadian phase of the retinal clock in vitro is still lacking. In photobiology, this is an essential prerequisite to enable meaningful comparisons between findings from different studies and to replicate experimental conditions. Our observations showed that the trough and the peak of the first PER2::Luc oscillation consistently occurred around circadian time (CT) 8 and CT20 of the circadian cycle (respectively, CT 7.65 ± 1.33 and CT 19.94 ± 1.55; mean ± SD; *n* = 42; S1 Fig). However, when explants were removed from the incubator for exposure to light (the method generally employed for light exposures), this induced random, robust advances or delays of the phase for each individual retinal explant (S2 Fig). Similar problems related to displacement have previously been shown for other in vitro cultures [46]. To avoid biases due to these artifactually induced phase shifts resulting from physical displacement, we developed a new light-emitting diode (LED)-based light delivery apparatus embedded within the Lumicycle (see Materials and methods). This procedure allowed for an accurate, artifact-free standard protocol to assess the photic dose-response properties (duration, irradiance) of the retinal clock.

**Fig 1:**
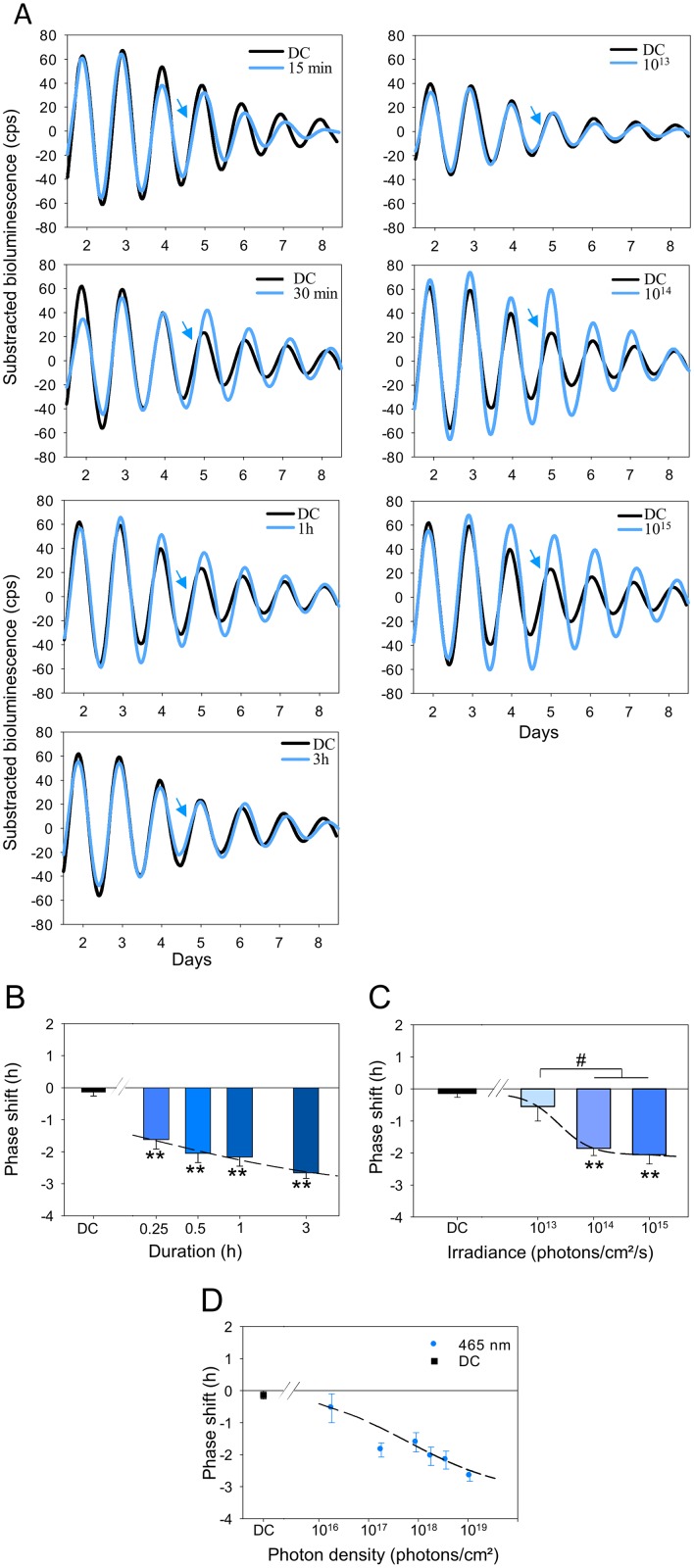
Temporal and irradiance responses for light-induced phase shifts of the retinal clock at 465 nm in the WT *Per2*^*Luc*^ mice. A. Representative PER2::Luc bioluminescence traces of retinal explants exposed to different durations (0.25, 0.5, 1, and 3 h; blue lines, left panel) and irradiances (10^13^, 10^14^, and 10^15^ photons/cm^2^/s; blue lines, right panel) compared to retinas not exposed to light (DC; black lines). The blue arrow indicates the time of the stimulation (CT16). B, C, D. Mean light-induced phase shift after a 465-nm light stimulation at CT16 of different durations with a constant irradiance (10^15^ photons/cm^2^/s) (B), at different irradiances with a constant duration (0.5 h) (C), and as a function of total photon density (D). Bars represent mean ± SEM (DC: *n* = 17; 0.25 h: *n* = 4; 0.5–3 h: *n* = 6–7; 10^13^–10^15^ photons/cm^2^/s: *n* = 5–7). *Represents statistically significant differences with the DC and ^#^statistical differences between different light conditions. **P < 0.001, ^#^*P* < 0.01. The data used to make this figure can be found in S1 Data. CT, circadian time; DC, dark control; PER2::Luc, PERIOD2::Luciferase; WT, wild-type.

Phase-shift properties of PER2::Luc wild-type (WT) retinas were first analyzed using 465 nm monochromatic light of different durations (0.25, 0.5, 1, or 3 h), at a constant irradiance (1 x 10^15^ photons/cm^2^/s), and subsequently at different irradiances for a fixed duration (0.5 h) at CT16. We observed that exposures to 465 nm light from 15 min to 3 h are sufficient to induce significant phase delays (15 min: −1.62 ± 0.30 h; 30 min: −2.05 ± 0.29 h; 1 h: −2.17 ± 0.28 h and 3 h: −2.67 ± 0.17 h; *P* < 0.001) in comparison to dark control retinas (dark control [DC]: −0.13 ± 0.13 h; Fig 1A, left panel, and Fig 1B). The slope of the stimulus 4-parameters curve (Naka–Rushton fit, Fig 1B) is steep, resulting in a narrow stimulus-duration range with a half maximum response at 0.51 h. In order to verify that the duration of culture and of light exposure did not affect the amounts of photopigments at the end of the experiment, levels of opsin mRNAs (MW and SW opsins, rhodopsin, melanopsin, and neuropsin) were quantified from the retinal explants (S3 Fig). We observed no differences in the relative expressions of the opsins between stimulated and DC retinas.

Using a 30-min duration (half-saturation value) of 465-nm light exposures to establish an irradiance-response curve, we found that 1 x 10^13^ photons/cm^2^/s was insufficient to induce a significant delay (−0.55 ± 0.45 h; *P* = 0.22; Fig 1A, right panel, and 1C), whereas higher irradiances (10^14^–10^15^ photons/cm^2^/s) produced significant phase delays (respectively, −1.73 ± 0.22 h and −2.05 ± 0.29 h; *P* < 0.01; Fig 1C). The slope of the irradiance-response curve again was steep, with an irradiance of 2.38 x 10^13^ photons/cm^2^/s necessary to induce a half maximum phase shift. A classical property of the circadian system is the ability to integrate photon number over time [47–50]. For the retina, plotting response amplitude in relation to the total number of photons yields a coherent photon dose-response function with a half maximum response at 5.53 x 10^17^ photons/cm^2^ and saturation of the response above 3.5 x 10^19^ photons/cm^2^ (Fig 1D).

### The retinal clock in WT *Per2*^*Luc*^ mice can be phase shifted by a wide range of visible wavelengths

**Fig 2:**
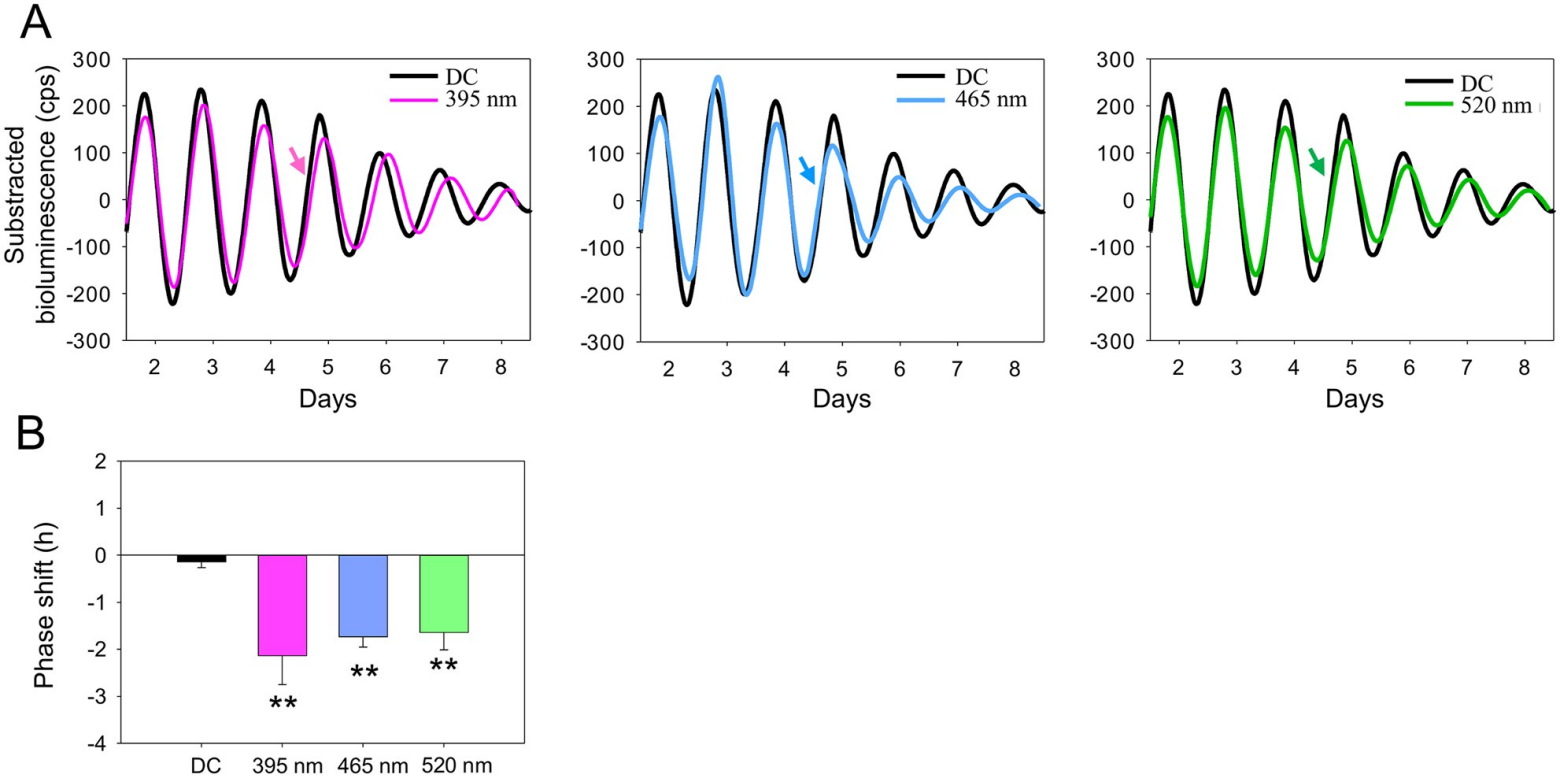
Different wavelengths of light induce a similar phase shift of the retinal clock in the WT *Per2*^*Luc*^ mice. A. Representative PER2::Luc bioluminescence traces of retinal explants exposed to 395 nm (purple line), 465 nm (blue line), or 520 nm (green line) compared to DC (black lines). Arrows indicate CT16, the time of the stimulation. B. Mean light-induced phase shift after a light stimulation (30 min, 10^14^ photons/cm^2^/s) at these three wavelengths. Bars represent mean ± SEM (DC: *n* = 17; 395–520 nm: *n* = 5–8). Statistical differences with DC are indicated by **P < 0.001. The data used to make this figure can be found in S1 Data. CT, circadian time; DC, dark control; PER2::Luc, PERIOD2::Luciferase; WT, wild-type.

Based on the optimal parameters of duration and irradiance, PER2::Luc retinal explants were exposed to equal quanta of monochromatic light (30 min, 1 x 10^14^ photons/cm^2^/s) of different wavelengths (395, 465, and 520 nm) at CT16. The choice of the three wavelengths was based on the peak photoreceptors sensitivities in the WT mouse (SW-cone opsin, λ_max_ = 360 nm; OPN5, λ_max_ = 370 nm, melanopsin, λ_max_ = 479 nm; rhodopsin, λ_max_ = 498 nm and MW-cone opsin, λ_max_ = 508 nm; S4A Fig; [37,42,51]). Significant phase delays of PER2::Luc are observed at 395 nm (−2.13 ± 0.62 h), 465 nm (−1.73 ± 0.22 h), and at 520 nm (−1.63 ± 0.38 h) compared to the DC (−0.13 ± 0.13 h, *P* < 0.001; Fig 2). The phase delays induced at 395, 465, and 520 nm were not significantly different from each other, suggesting that the irradiances used were at saturating levels. In addition, since the stimulation at 520 nm corresponds to a more than 5 log unit decrease in SW opsin and OPN5 sensitivities (S4B Fig), it appears unlikely that OPN5 alone can account for the light-induced phase shifts of the retinal clock in the visible range of the spectrum, suggesting a putative role of rods, MW cones, and/or melanopsin.

### Light-induced phase shift of the retinal clock is abolished in rodless *Nrl*^−/−^ mice

**Fig 3:**
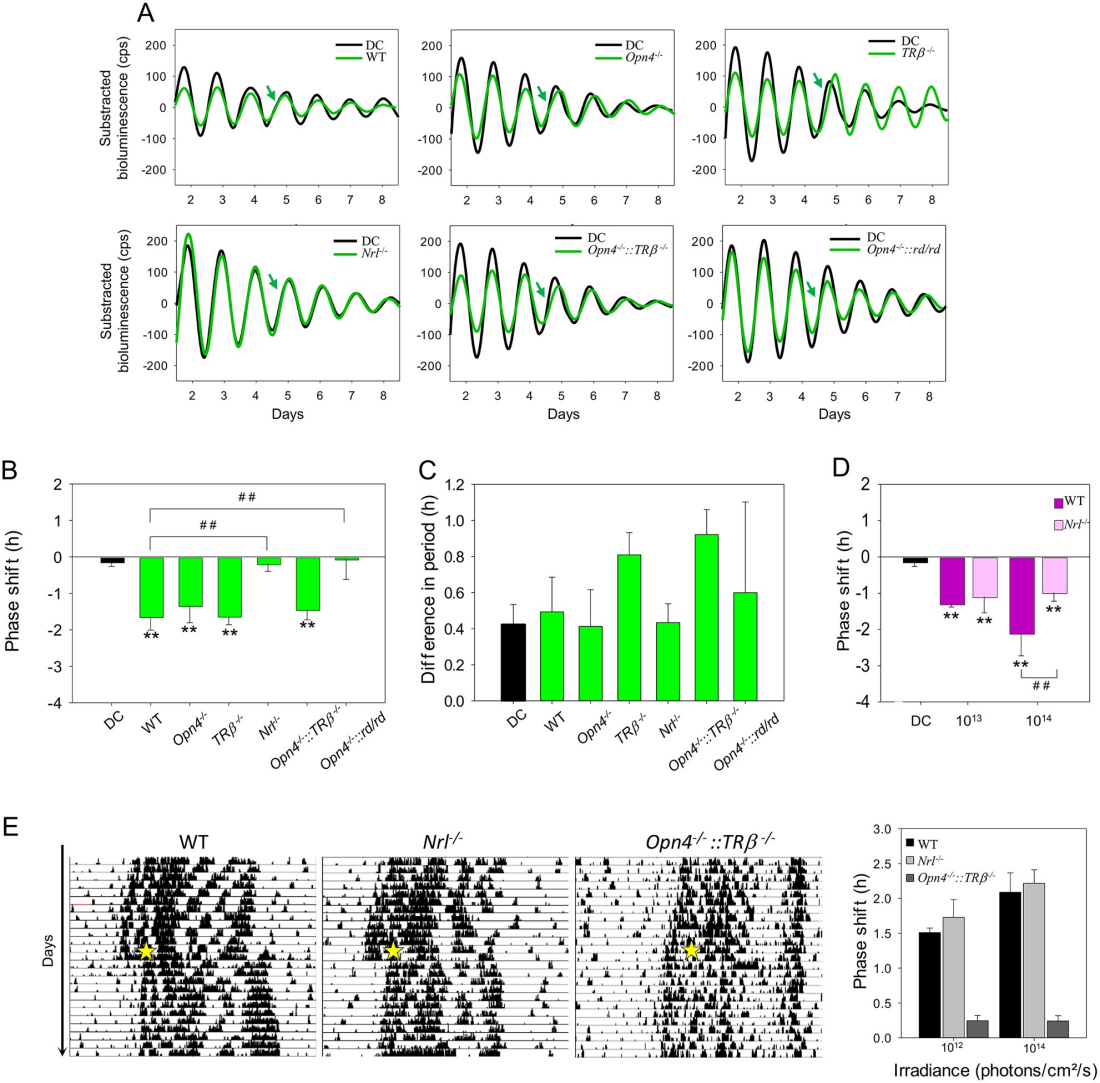
Light response of the retinal clock in WT *Per2*^*Luc*^ and photoreceptor-deficient (*Opn4*^−/−^::*Per2*^*Luc*^, *TRβ*^−/−^::*Per2*^*Luc*^, *Nrl*^−/−^::*Per2*^*Luc*^, *Opn4*^−/−^::*TRβ*^−/−^::*Per2*^*Luc*^, and *Opn4*^−/−^:: *rd/rd*::*Per2*^*Luc*^) mice. A. Representative PER2::Luc bioluminescence traces of retinal explants from *Per2*^*Luc*^ and photoreceptor-deficient mice exposed to 520 nm (30 min, 10^14^ photons/cm^2^/s; green line) compared to DC (black line). Green arrows indicate CT16, the time of the stimulation. B. Mean light-induced phase shift in homozygous genotypes. C. Difference in the endogenous period before and after the light stimulation. A positive value corresponds to a lengthening of the period. Bars represent mean ± SEM (DC: *n* = 17; WT: *n* = 8; for homozygous photoreceptor deficient mice: *n* = 3–8). D. Mean light-induced phase shift in the WT and *Nrl*^−/−^::*Per2*^*Luc*^ following a 395-nm light stimulation (30 min, 2.8 x 10^13^ and 2.8 x 10^14^ photons/cm^2^/s; DC: *n* = 17; WT: *n* = 8; *Nrl*^−/−^::*Per2*^*Luc*^: *n* = 10). *Represents statistical differences with the DC and ^#^statistical differences between different genotypes. *P < 0.01, **, or ^# #^*P* < 0.001. E. Representative actograms of locomotor activity of a WT, a *Nrl*^−/−^::*Per2*^*Luc*^, and an *Opn4*^−/−^::*TRβ*^−/−^::*Per2*^*Luc*^ mouse exposed to 15 min pulses of 530 nm monochromatic light at CT16 (2.8 x 10^14^ photons/cm^2^/s; yellow star) and mean light-induced phase shift after a light stimulation at 2.8 x 10^12^ and 2.8 x 10^14^ photons/cm^2^/s. No statistical difference was observed between WT and *Nrl*^−/−^::*Per2*^*Luc*^ mice, whereas *Opn4*^−/−^::*TRβ*^−/−^::*Per2*^*Luc*^ mice exhibit a reduced phase shift at both irradiances. Bars represent mean ± SEM (WT: *n* = 6; *Nrl*^−/−^::*Per2*^*Luc*^: *n* = 5; *Opn4*^−/−^::*TRβ*^−/−^::*Per2*^*Luc*^: *n* = 3). The data used to make this figure can be found in S1 Data. CT, circadian time; DC, dark control; *Nrl*, retina-specific leucine zipper protein; *Opn4*, melanopsin; PER2::Luc, PERIOD2::Luciferase; *TRβ*, thyroid hormone receptor beta; WT, wild-type.

To determine the photoreceptors involved in the phase-shift response of the retinal clock, we used 520-nm stimulations to rule out possible contributions of SW cones and OPN5. However, at this wavelength, MW cones, rods, and ipRGCs have relatively similar spectral sensibilities, precluding determination of their relative contributions in the WT mouse. We thus backcrossed mouse models that are deficient for each of these photoreceptors with the *Per2*^*Luc*^ mice to obtain rodless (*Nrl*^−/−^::*Per2*^*Luc*^), MW coneless (*TRβ*^−/−^::*Per2*^*Luc*^), and melanopsin knockout (*Opn4*^−/−^::*Per2*^*Luc*^) models. A “rod-only” (*Opn4*^−/−^::*TRβ*^−/−^::*Per2*^*Luc*^) model was obtained by crossing *Opn4*^−/−^::*Per2*^*Luc*^ and *TRβ*^−/−^::*Per2*^*Luc*^ mice. *Opn4*^−/−^::*Per2*^*Luc*^, *TRβ*^−/−^::*Per2*^*Luc*^, and *Opn4*^−/−^::*TRβ*^−/−^::*Per2*^*Luc*^ mice show a similar phase shift (respectively, −1.33 ± 0.47 h; −1.63 ± 0.23 h and −1.44 ± 0.28 h; Fig 3A and 3B) following 30 min exposure to 1 x 10^14^ photons/cm^2^/s at 520 nm compared to WT *Per2*^*Luc*^ mice (−1.46 ± 0.30 h; *P* = 0.38, *P* = 0.51, and *P* = 0.34). In contrast, the absence of rods in the *Nrl*^−/−^::*Per2*^*Luc*^ model totally abolished the light-induced phase shift at 520 nm (−0.18 ± 0.21 h, *P* < 0.01). Furthermore, the absence of cones, rods, and melanopsin in the *Opn4*^−/−^::*rd/rd*::*Per2*^*Luc*^ mice prevented any light-induced phase shift of the retinal clock (−0.05 ± 0.56 h, *P* < 0.01). To eliminate the possibility that the differences in the phase shift could be related to a light-induced change in the period, we compared periods before and after the stimulation in retinas and in the DC and found no differences between genotypes (Fig 3C). The same experiments have been performed on heterozygous mice of each genotype, and similar results were obtained (S5A and S5B Fig). Taken together, these results suggest that rod input alone is required to shift the retinal clock in the visible part of the spectrum. We then assessed whether a phase shift of *Nrl*^−/−^::*Per2*^*Luc*^ retinal explants could be obtained in the UV part of the spectrum. At 1 x 10^13^ photons/cm^2^/s (30 min, 395 nm), we found a significant and similar phase delay in both WT *Per2*^*Luc*^ and *Nrl*^−/−^::*Per2*^*Luc*^ retinal explants (respectively, −1.29 ± 0.09 h and −1.09 ± 0.45 h) by comparison with the DC retinas (*P* < 0.01; Fig 3D). In the WT mouse, the phase delay obtained at 395 nm is significantly increased from the DC retinas, whereas the same equal quanta stimulation at 465 nm did not induce a significant phase shift (Fig 1C). Using an increased irradiance at 395 nm (1 x 10^14^ photons/cm^2^/s; 30 min), we found a significantly reduced phase shift (−0.98 ± 0.24 h) in the *Nrl*^−/−^::*Per2*^*Luc*^ compared to that of WT *Per2*^*Luc*^ retinal explants (−2.13 ± 0.62 h; *P* < 0.05). The residual phase shift in the UV suggests a possible involvement of SW cones and/or OPN5 in addition to rods.

To ascertain if the *Nrl*^−/−^::*Per2*^*Luc*^ mouse presents a global deficit in the light response of the circadian system, we examined the amplitude of locomotor activity phase shifts induced by a 15-min pulse of 530 nm monochromatic light at two different irradiances (2.8 x 10^12^ and 2.8 x 10^14^ photons/cm^2^/s; Fig 3E). No significant differences in the magnitude of the phase shift and the pattern of locomotor activity were observed between *Nrl*^−/−^::*Per2*^*Luc*^ and WT mouse at both irradiance levels used. By contrast, the light-induced phase shift is dramatically reduced at both irradiances in the *Opn4*^−/−^::*TRβ*^−/−^::*Per2*^*Luc*^ mice, which otherwise retained light-induced phase shift of the retinal clock (Fig 3B and 3E).

Finally, we assessed whether invalidation of photopigments in the different mouse strains altered the endogenous functioning of the retinal clock. Our results show a role of melanopsin and/or MW cones since a significant shortening of the endogenous period was observed in *Opn4*^−/−^::*Per2*^*Luc*^ (23.93 ± 0.15 h; *P* < 0.001), *TRβ*^−/−^::*Per2*^*Luc*^ (23.71 ± 0.09 h; *P* < 0.001), *Opn4*^−/−^::*TRβ*^−/−^::*Per2*^*Luc*^ (23.82 ± 0.01 h; *P* < 0.001), and *Opn4*^−/−^::*rd/rd*::*Per2*^*Luc*^ (24.21 ± 0.2 h; *P* < 0.05) mice compared to WT *Per2*^*Luc*^ (24.69 ± 0.08 h; S5C Fig).

## Discussion

In this in vitro study, we provide the first in-depth analysis of irradiance and duration light responses for the retinal clock and confirm that, similar to the circadian system, the dose-response curve exhibits a typical reciprocity function in terms of the total number of photons required to produce a phase shift. Our results also show that rods are required for light-induced phase shifts of the murine retinal clock in the visible region of the light spectrum and reveal putative additional recruitment of SW cones and/or OPN5 at shorter UV wavelengths. We also provide evidence that melanopsin and MW cones are involved in the regulation of the endogenous period of the retinal clock.

### Light responses of the retinal clock to duration and irradiance

Comparison of photoreceptor spectral absorptions with the relative sensitivity of evoked responses is a critical strategy for identification of the photopigments mediating non-image forming (NIF) responses to light in rodents [52]. Light entrainment of the retinal clock is gated in a phase-specific manner, as in the SCN, with maximum phase delays occurring at CT16 and phase advances during the late subjective night [23–25].

**Fig 4:**
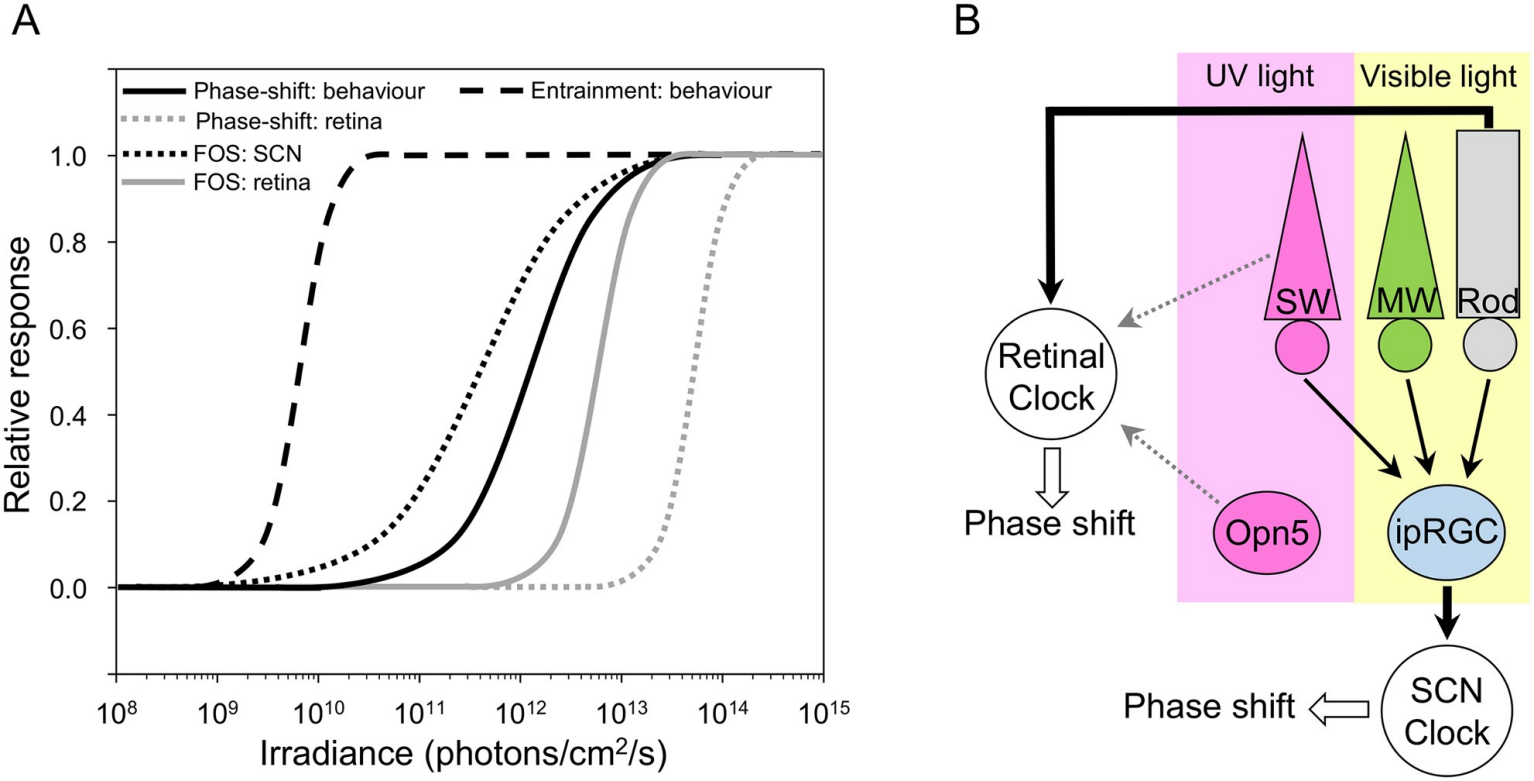
The light-response of SCN and retinal clocks is different and involves distinct photoreceptors. A. Irradiance-response curves of different light responses in SCN and retinal clocks. This figure summarizes results of the present and previous studies of behavioral entrainment, phase shift of locomotor activity and PER2::Luc rhythms in the retina, and FOS expression in the SCN and retina. Only studies using monochromatic light (465–520 nm) are presented to provide valid comparisons in terms of irradiance. The irradiance threshold of FOS induction in the retina [49] is lower than the threshold of the retinal clock (present study). Light responses of the SCN including behavioral entrainment [54] and phase shift [18,20,53,54,56] show even lower thresholds compared to the retinal clock (present study). B. The phase-shifting responses of retinal and SCN clocks rely upon distinct photoreceptors. ipRGCs that receive inputs from both rods and cones (MW and SW) act as the primary sensory conduit mediating NIF responses to light (thick black arrows), including entrainment and light-induced phase shift of the SCN. The phase-shifting response to light of the retinal clock is dependent on rod input in the visible spectrum and may involve additional recruitment of SW cones and/or OPN5-expressing cells at shorter wavelengths (grey arrows). ipRGC, intrinsically photosensitive melanopsin-containing retinal ganglion cell; MW, middle-wavelength; NIF, non-image forming; OPN5, neuropsin; PER2::Luc, PERIOD2::Luciferase; SCN, suprachiasmatic nucleus; SW, short-wavelength.

The dose-response function for eliciting a phase shift and reciprocity, core properties of the circadian system, have not been previously evaluated for the retinal clock. These properties translate the ability to integrate photic input over a relatively long period of time, ranging from a few seconds to several hours, and to respond proportionally to the total energy of the stimulus [47–50]. Compared to previous studies in the retina [23–25], we find that relatively shorter duration exposures (15 min) at lower irradiance levels (1 x 10^14^ photons/cm^2^/s) are sufficient to induce a phase delay of PER2::Luc signal. In particular, the studies by Buhr and colleagues used 3-h light exposures at 1 x 10^15^ photons/cm^2^/s [24, 25]. This value in terms of total photon number is 2 log units higher than the amount required to induce a phase shift in our study and is in the range of saturating amounts. The stimulus irradiance threshold for eliciting a retinal phase shift is nevertheless relatively high (>10^13^ photons/cm^2^/s, present study) compared to the energy required for a behavioral phase shift (approximately 10^10^–10^11^ photons/cm^2^/s, [18,20,53,54]). Fig 4A shows the differences in sensitivity for phase-shifting and entrainment responses of retinal and SCN clocks to light between 465–520 nm from our and other laboratories [18,20,22,49,53–56]. Furthermore, the retinal clock appears unable to integrate light energy for durations longer than 30 min. This response resembles the pattern of light-induced FOS expression in the retina that increases sharply at a relatively high level of irradiance and long duration (Fig 4A, [49]). The difference in light sensitivity between retinal and SCN clocks suggests clock-specific tuning of light responses in each system that may be related to different integration and/or feedback mechanisms in these two clocks, downstream from the photoreceptor level.

### Rods are required for in vitro light-induced phase shift of the retinal clock

A potential role of rods, cones, and ipRGCs in the light response of the retinal clock has recently been challenged by two studies from Buhr and colleagues claiming that none of these photoreceptors are involved in entrainment of the retinal clock and that OPN5, a UV-sensitive retinal opsin, is the sole photopigment involved [24,25]. Our findings show phase delays of PER2::Luc oscillation using similar quanta of 395, 465, or 520 nm light, demonstrating that the retinal clock is capable to respond across a broad range of visible wavelengths. Since the spectral sensitivity of OPN5 is attenuated by more than 5 log units at 520 nm (S4B Fig), a robust response at this wavelength strongly argues against an exclusive mediation of light-induced phase shifts by OPN5. Furthermore, even at the short wavelength (417 nm) employed by Buhr and colleagues [25], it is difficult to rule out the possibility that the long-duration and high-irradiance exposures applied at this wavelength [25] could also activate rods, MW cones, and/or ipRGCs.

Since MW cones, rods, and ipRGCs have largely overlapping spectral sensitivities and are thus difficult to completely isolate in the WT mice using spectral stimulation strategies, we used *Per2*^*Luc*^ mouse models deficient in different photoreceptor classes (*Nrl*^−/−^::*Per2*^*Luc*^; *TRβ*^−/−^::*Per2*^*Luc*^, *Opn4*^−/−^::*Per2*^*Luc*^, *Opn4*^−/−^::*TRβ*^−/−^::*Per2*^*Luc*^, and *Opn4*^−/−^::*rd/rd*::*Per2*^*Luc*^). The *Nrl* and *TRβ* genes are essential for photoreceptor development. In *TRβ*^−/−^ knockout mouse, MW opsin is not expressed, and all cones express SW opsin [55,57]. *Nrl* encodes a transcription factor that is essential for rod development [58–60]. When *Nrl* gene is knocked out, a complete absence of rods is observed, as revealed by histology, immunocytochemistry, electrophysiology, and gene expression analysis. Rod progenitor cells differentiate into SW cones [59,61,62], and this mouse is widely used as a cone-only model in vision [63]. We found no change in the relative expression of OPN5 and a slight increase in melanopsin and MW opsin mRNAs (S6 Fig). In addition, behavioral phase shifts in WT and *Nrl*^−/−^ mice are similar, indicating a dichotomy of the light response between retinal and central clocks. The use of these models suggests that rods but neither MW cones or melanopsin are required for in vitro light-induced phase shift of the mouse retinal clock. In agreement, Buhr and colleagues [24,25] showed in WT mice (in which rods are conserved) a large phase shift of more than 3 h using 475-nm stimulus in the rod sensitivity region of the visible light spectrum.

The involvement of rods at the relatively high irradiance levels employed here appear paradoxical compared to their sensitivity range for visual responses at low scotopic light levels. However, rods have a wide response range and can drive dopaminergic cell responses in the retina, pupillary constriction, and behavioral entrainment not only under dim light levels but also at higher light levels within the sensitivity range of cones [64–66]. This response appears to be mediated through rod–cone pathways, involving gap junctions between rods and cones [65]. As a preliminary observation, retinal explants from *Per2*^*Luc*^ mice that were exposed to 520 nm light in the presence of carbenoxolone (CBX), a general gap junction blocker, fail to exhibit a phase shift compared to DC (CBX: 0.5 ± 0.04 h; *n* = 4; *P* = 0.21). These data are thus consistent with the idea that rods have the capacity to elicit phase shifts to light at high irradiances for both retinal and SCN clocks (Fig 4B).

Since rods are involved, the relatively high threshold necessary to obtain a phase shift of the retinal clock is unexpected. Indeed, a 30-min exposure of 1 x 10^13^ photons/cm^2^/s of 465 nm light is insufficient to induce a phase shift. Compared to rod-driven visual responses, thresholds for NIF responses are, in general, relatively high and can vary in sensitivity by several log units, depending on the response (Fig 4A, [18,20,22,49,53–56]). Even in WT mice, the threshold to elicit a behavioral phase shift is in the mesopic range (approximately 10^10^–10^11^ photons/cm^2^/s [18,20,53–56], Fig 4A), well above the sensitivity of rods [67]. In support of a difference in light sensitivity between retinal and SCN clocks, we also found differences in their phase-shifting response in the *Nrl*^−/−^::*Per2*^*Luc*^ and the *Opn4*^−/−^::*TRβ*^−/−^::*Per2*^*Luc*^, suggesting a clear dichotomy in their light response.

The discrepancy between the current study and those of Buhr and colleagues may be related to methodology and the responses studied [25]. A first issue is related to the variable artifactual phase shift caused by physical displacement of the retinal explants, an effect also previously shown in cultured retinal pigment epithelium (RPE) [46]. The use of long light durations (3 h) by Buhr and colleagues [24,25] may, to some extent, mask the effect of the physical displacement on the phase of the retinal clock. Secondly, these authors mainly used an entrainment paradigm (9L:15D, [24,25]), whereas we (present study) and Ruan and colleagues examined the acute effect of short-duration light pulses on the phase of the retinal clock [23]. Moreover, the failure of WT retinal explants to entrain to a light:dark (L:D) cycle at 530 nm [25] is puzzling, since significant phase shifts have been demonstrated using, respectively, 520 nm or broadband while light (present study, [23]).

Our data show a residual sensitivity to light at 395 nm in rodless mice, suggesting that OPN5 and/or SW cones may contribute to the phase shift (Fig 3D). Buhr and colleagues attribute the phase shift of the retinal clock in SW knockout mice at 417 nm to OPN5 [25]. However, the use of long-duration and high-irradiance light does not exclude a rod contribution since the spectral sensitivity of rods is only slightly reduced compared to that of OPN5 at this wavelength. High light levels in in vitro explant cultures that lack photoprotective RPE and light absorption by the lens can also provoke phototoxic cell damage and other deleterious effects [68,69]. The RPE plays an important role in the maintenance of the health and function of photoreceptors. In particular, rhodopsin and cone opsin pigments require a continuous supply of visual chromophore to maintain photosensitivity in bright light. This is carried out by multistep enzyme pathways in the RPE and Müller cells. We designed our retinal explant culture without the RPE for three main reasons: firstly, the RPE also contains a circadian clock with a rhythmic expression of PER2::Luc [46,70–72] that may interfere with the signal emitted by the retina alone. Secondly, Kaylor and colleagues recently demonstrated in vivo and in vitro that rhodopsin may regenerate in photoreceptor membranes in the absence of the RPE [73]. Finally, we were careful in our strategy to use a single light stimulation in each retinal explant to avoid issues of photopigment depletion (S3 Fig).

The effect of photoreceptor/photopigment loss on the endogenous period of the retinal clock may have different explanations. The retinal clock is composed of a network of multiple strongly coupled circadian oscillators located within distinct cellular layers [16,30,45,74]. Photoreceptors (cones and ipRGCs) have also been shown to contain a cellular circadian clock (for review, see [2]), with the notable exception of rods. All of the mouse models examined in the present study (*Opn4*^−/−^::*Per2*^*Luc*^, *TRβ*^−/−^::*Per2*^*Luc*^, *Opn4*^−/−^::*TRβ*^−/−^::*Per2*^*Luc*^, and *Opn4*^−/−^::*rd/rd*::*Per2*^*Luc*^*)* exhibit shortening of the endogenous period, except the *Nrl*^−/−^::*Per2*^*Luc*^ mouse model. This effect on the endogenous period of the retinal clock may be related to the absence of one of these cellular oscillators from a complex tightly coupled network leading to a modification of the endogenous period at the level of the entire retina. In agreement with this hypothesis, we did not observe any impact of the absence of rods on the period of the retinal clock in the *Nrl*^−/−^::*Per2*^*Luc*^ mice. In addition, it cannot be excluded that the period changes might be secondary to developmental effects induced by the absence of a photoreceptor/photopigment.

In conclusion, our findings reveal that the absence of rods but not of melanopsin or MW cones totally prevented a light-induced phase shift in the visible spectrum and suggest additional recruitment of SW cones and/or OPN5 at shorter UV wavelengths. A putative role of OPN5 in humans and nonhuman primates is, however, questionable since, in contrast to UV transmittance of the mouse lens, UV wavelengths are effectively blocked out by the human/primate lens [68,75–81]. Furthermore, while the first study reported the expression of OPN5 in human retinas [36], recent works do not detect this opsin both in human [37] and in nonhuman primate retinas [70]. Finally, we show a clear dichotomy in the light response between retinal and SCN clocks, highlighting the need for a comprehensive understanding of the neural circuits involved in the light response of the retinal clock.

## Materials and methods

### Ethics statement

All animal procedures were in strict accordance with current national and international regulations on animal care, housing, breeding, and experimentation and were approved by the regional ethics committee CELYNE (C2EA42-13-02-0402-005). All efforts were made to minimize suffering.

### Animals

Mice were housed in a temperature-controlled room (23 ± 1 °C) under 12 h light/12 h dark cycle (12L/12D, light intensity around 200 lux), with food and water ad libitum. *Per2*^*Luc*^ mice [82] and several photoreceptor-deficient mice were used: *TRβ*^−/−^ lacking MW opsin [55,83], *Opn*_*4*_^−/−^ knockout for melanopsin [20], and *Nrl*^−/−^, characterized by the complete loss of rods and an increased number of SW cones [59]. All photoreceptor-deficient mice were bred with the *Per2*^*Luc*^ mice to obtain *TRβ*^−/−^::*Per2*^*Luc*^, *Opn*_*4*_^−/−^::*Per2*^*Luc*^, and *Nrl*^−/−^::*Per2*^*Luc*^ mice. *TRβ*^−/−^::*Per2*^*Luc*^ and *Opn*_*4*_^−/−^::*Per2*^*Luc*^ were then crossed together to obtain *Opn*_*4*_^−/−^::*TRβ*^−/−^::*Per2*^*Luc*^ mice. The *Opn4*^−/−^::*rd/rd*::*Per2*^*Luc*^ mice were obtained from Dr. Van Gelder and Dr. Buhr. All lines were maintained on a C57BL/6J background. We used female and male mice in all experiments. All mice were used between 2–4 months old, except the *Nrl*^−/−^::*Per2*^*Luc*^ model, which were used at 4 weeks before the onset of apoptotic degeneration [84]. The *Opn4*^−/−^::*rd/rd*::*Per2*^*Luc*^ mice were aged >200 days. To verify that photoreceptor degeneration (rods and cones) was complete, light entrainment of the rhythm of locomotor activity of each animal was analyzed. Only animals exhibiting a free-running locomotor rhythm were used.

### Retinal explant culture and bioluminescence recording

Mice were killed by cervical dislocation 1 h before light offset (Zeitgeber Time 11 or ZT11), except the *Opn4*^−/−^::*rd/rd*::*Per2*^*Luc*^ mice that were euthanized at CT11 according to their behavioral locomotor rhythm. Eyes were enucleated and placed in Hank’s balanced salt solution (HBSS; Invitrogen) on ice. Retinas were gently isolated from the rest of the eye cup and flattened ganglion cell layer upon a semipermeable (Millicell) membrane in a 35-mm culture dish (Nunclon) containing 1.2 mL Neurobasal-A (Life Technologies) with 2% B27 (Gibco), 2 mM L-Glutamine (Life Technologies), and 25 U/mL antibiotics (Penicillin/Streptomycin, Sigma), incubated at 37 °C in 5% CO_2_ for 24 h. From this step on, all manipulations of explants were performed under dim red light. After 24 h, at the projected ZT12, retinas were transferred to 1.2 ml of 199 medium (Sigma), supplemented by 4 mM sodium bicarbonate (Sigma), 20 mM D-glucose (Sigma), 2% B27, 0.7 mM L-Glutamine, 25 U/mL antibiotics (Penicillin/Streptomycin, Sigma), and 0.1 mM Luciferin (Perkin). Culture dishes were sealed and then placed in a Lumicycle (Actimetrics, Wilmette, IL, United States of America) to record the global emitted bioluminescence. For blocking gap junction, 100 μM CBX was added to 199 medium. PER2::Luc bioluminescence was analyzed using Lumicycle Analysis software (Actimetrics, Wilmette, IL, USA).

### Determination of the biological time of the retinal clock in vitro

All retinal explants were dissected at ZT11 and cultured just before light offset (ZT12). The projected ZT12, at which medium was changed and recording started, was then considered as CT12 and used as a time reference (S1A Fig). The time of occurrence of the trough and the peak of the first complete PER2::Luc oscillation were determined by using this CT12 reference and by correcting time for the endogenous period (S1B Fig). The phase of the peak was then used to calculate the timing of the following CT16 to apply light stimulation or physical displacement.

### Physical displacement effects on the retinal clock phase

The classical procedure commonly employed to assess light-induced phase shifts involves the transfer of the cultured tissue from the Lumicycle into a light-stimulation chamber. To evaluate the effects of displacement of retinal culture dishes on the phase of PER2::Luc, we first established a 4-day baseline bioluminescence signal for each sample in the Lumicycle (Actimetrics), and retinal explants were then cautiously transferred to a nearby incubator in a light-proof, insulated chamber at CT16. Subsequently, the tissue was returned to the Lumicycle, and the phase shift was measured. Each retinal explant was submitted to three successive movements, each separated by a medium change.

### Light delivery apparatus

To avoid effects of displacement, we developed a new light-delivery apparatus embedded within the Lumicycle. This device is composed of an opaque matrix that fits to the shape of the five exposed dishes on the turntable and by a black cylinder reflective white inside containing the LEDs (Super Bright LEDs). To avoid any light diffusion to the photomultiplier tubes during the light stimulation, the bottom edge of the cylinder was sealed to the contours of the matrix with light impermeable seals inside the Lumicycle. Temperature was monitored by placing a temperature data logger (HOBO data logger, ONSET) inside the light delivery apparatus, at the level of the retinal explants. The temperature change occurring during light stimulation is 0.57 ± 0.01 °C. Retinas placed on the opposite side of the Lumicycle during the light stimulations were used as DCs. To exclude that the temperature change in the incubator due to the heat from the light source is responsible for the phase shift, an additional control was included. Using the same apparatus, we applied a similar light stimulation (520 nm, 30 minutes, 10^14^ photons/cm^2^/s) with an opaque filter inserted between the LEDs and the retinal explants and completely blocks any light transmission to the retinal cultures. The change in temperature (0.59 °C ± 0.01 °C) is identical to that observed without the opaque filter (0.57 °C ± 0.01 °C). Under these conditions, no significant phase delay of PER2::Luc oscillations of the retinal explants was observed (−0.52 ± 0.19 h, *n* = 4, *P* = 0.13).

### Phase shifting response and data analysis

Raw bioluminescence data were detrended using a 25-h running average and smoothed with a 3-h running average method. The phase and the period were determined by using best-fit sine wave function (sin fit) of the Lumicycle Analysis software. Using this function, the phases (pre- and poststimulation) are determined as the maximum of the oscillation. The phase of the prestimulation rhythm (phase of the third oscillation before the light stimulation) was used to predict the phase of the PER2::Luc oscillation (phase of the third oscillation before the light stimulation plus two values of the endogenous period) if no light stimulation (or mechanical displacement) was applied (predicted phase). The calculation of the phase shift is based on the difference between the predicted phase and the observed phase (phase of the first complete oscillation after the light stimulation/displacement) in the same retinal explant. The observed phase is also determined by the best sine wave function (sin fit function) on three complete oscillations after light stimulation/displacement. The circadian period (pre- and poststimulation) are determined on the 3 days, respectively, before and after light stimulation. The phase shifts were calculated by the experimenter and by a person blind to the different light/genotype conditions. The goodness of fit was determined and was between 93.3% and 97.5%, with an average of 95.93% ± 0.29%.

According to the experiment, retinal explants are exposed to different durations (0.25, 0.5, 1, and 3 h) and irradiances (10^13^, 10^14^, and 10^15^ photons/cm^2^/s) of 465 nm monochromatic light. Light stimulations were done at CT16 since previous data has shown that light maximally phase delays the retinal clock at this CT time [25]. Thereafter, we used constant irradiance and duration (10^14^ photons/cm^2^/s, 30 min) to study the effect of different wavelengths (395, 465, and 520 nm) using bright LED light sources (Superbrightleds; S4C Fig). Data from the irradiance, duration, and total number of photons were fit with a four-parameter logistic equation using a modified form of the Naka–Rushton equation previously described [49,85]. Radiometric measurements were made by using an International Light model IL1700 photometer (International Light Technologies) and a spectrophotometer (Specbos 1211, JETI).

### Quantitative RT-PCR

The relative expression of the different opsins was quantified in cultured WT *Per2*^*Luc*^ retinas (light stimulated and DC) and collected at the end of the duration experiment and in retinas from 4-week-old WT and *Nrl*^−/−^ mice. Cultured retinas were snap frozen at the trough after 10 days in culture, during which they had been exposed to 0.5, 1, or 3 h of light or no light (DC). Total RNA was extracted using Trizol reagent (Invitrogen) and reverse transcribed using random primers and MMLV Reverse Transcriptase (Invitrogen). Real-time reverse transcription PCR (RT-PCR) was then performed on a LightCycler system (Roche Diagnostics) using the light Cycler-DNA Master SYBR Green I mix. *Hprt* was used for internal standardization of target gene expression. The efficiency and the specificity of the amplification were controlled by generating standard curves and carrying out melting curves. Relative transcript levels of each gene were calculated using the second derivative maximum values from the linear regression of cycle number versus log concentration of the amplified gene. Primer sequences were *Hprt* sens ATCAGTCAACGGGGGACATA and reverse AGAGGTCCTTTTCACCAGCA, *SW opsin* sens CAGCCTTCATGGGATTTG and reverse GTGCATGCTTGGAGTTGA, *MW opsin* sens GCTGCATCTTCCCACTCAG and reverse GACCATCACCACCACCAT, *Rhodopsin* sens GCCACCACTCAGAAGGCAG and reverse GATGGAAGAGCTCTTAGCAAAG, *Melanopsin* sens TGCGAGTTCTATGCCTTCTG and reverse GGCACGTAGGCACTCCAAC, *Neuropsin* sens ACTATGCACCTGAGCCCTTC and reverse TGGCTGCTATGGATTCGACT, and *Per2* sens CCACACCTTGCCTCCGAAAATA and reverse ACTGCCTCTGGACTGGAAGA.

### Behavioral phase-shifting assay

Singly housed male WT *Per2*^*Luc*^, *Nrl*^−/−^::*Per2*^*Luc*^, and *Opn*_*4*_^−/−^::*TRβ*^−/−^::*Per2*^*Luc*^ mice (WT: *n* = 6; *Nrl*^−/−^::*Per2*^*Luc*^ and *Opn*_*4*_^−/−^::*TRβ*^−/−^::*Per2*^*Luc*^: *n* = 3–5) were first entrained in a 12L/12D cycle for 20 days. Subsequently, animals were maintained in constant darkness (DD) to examine the free-running period calculated by periodogram analysis using ClockLab software (Actimetrics). Phase shifts were studied using a single 15-min monochromatic light pulse (530 nm, half-bandwidth, 10 nm) at 2.8 x 10^12^ photons/cm^2^/s and 2.8 x 10^14^ photons/cm^2^/s applied at CT16. The stimulator (light source and chamber) has been described previously [49,55]. After the light pulse, animals were returned to their home cages, and activity was monitored in DD for an additional 15 days before the next light pulse. The magnitude of a light-induced phase shift was determined from the difference between the regression lines of the activity onsets before and after the light stimulation, extrapolated to the day following the light pulse. The transient responses on the 3–4 days immediately after the pulse were discounted [86].

### Retinal histology

Four-week-old WT and *Nrl*^−/−^ mice were killed by CO_2_ inhalation and decapitation. Whole eyes were dissected and immersion fixed in 4% paraformaldehyde in phosphate-buffered saline (PBS) overnight at 4 °C. Eyes were frozen, cut at 20-μm thickness, and mounted on glass slides. Retinal sections were hydrated in graded ethanol solutions (95%, 75%, 50%) for 30 s each. The retinal sections were then stained with 1% cresyl violet and dehydrated in graded ethanol solutions (50%, 75%, 95%, 100%).

### Statistical analysis

Normal distribution of the data and homogeneity of the variance were respectively tested using Shapiro–Wilk and the Levene’s test. Statistical analyses were performed using one-way ANOVA followed, when significant, (*P* < 0.05) by Fisher’s LSD posthoc test. Results are expressed as mean ± SEM.

## Supporting information

S1 Data Excel spread sheet containing, in separate sheets, data for Figs 1A–1D, 2A, 2B and 3A–3E, S1B, S2, S3, S4A–S4C, S5A–S5C and S6B Figs, and underlying raw values used to generate averages. (XLSX)

S1 Fig Determination of a marker of PER2::Luc oscillation. A. Schematic representation of the protocol used for the retinal explant culture and calculation of the circadian time of the oscillation. Retinal explants were dissected at ZT11 and cultured just before light offset (ZT12). The projected ZT12 is then considered as CT12 and used to predict the circadian time of the retinal clock in vitro. Arrowheads correspond to the trough and the peak of the first complete oscillation. B. The first trough (white circles) and peak (black circles) of PER2::Luc oscillation occur, respectively, at CT 7.65 ± 1.33 and CT 19.94 ± 1.55 (mean ± SD). Each circle on the same line represents the trough and the peak of the same retinal explant (*n* = 42). The data used to make this figure can be found in S1 Data. CT, circadian time; PER2::Luc, PERIOD2::Luciferase; ZT, zeitgeber time.
(TIF)

S2 Fig Physical displacement of tissue culture induces robust and random phase shifts of the retinal clock. For light-induced phase shift experiments of the retinal clock, the classical procedure involves the transfer of the cultured tissue into a light stimulator outside the Lumicycle. The effect of physical displacement on the phase of PER2::Luc expression was analyzed following three successive displacements of the culture dishes. We show for the same retinal explant a robust and random effect of displacement on the phase of PER2::Luc (advance or delay) that may simply result from a medium homogenization. Each symbol corresponds to an individual explant (*n* = 6). Bars represent the mean ± SEM. The data used to make this figure can be found in S1 Data. PER2::Luc, PERIOD2::Luciferase.
(TIF)

S3 Fig Effect of the duration of the light stimulation on the relative expression of opsins mRNA in retinal explants from *Per2*^*Luc*^ mice. Relative expression of opsins (MW opsin, SW opsin, rhodopsin, melanopsin, and OPN5) of 10-day-cultured retinas stimulated by different durations (0.5 h, 1 h, and 3 h; grey bars) at 465 nm was compared to DC retinas (black bars). Bars represent mean ± SEM (DC: *n* = 3; 0.5–3 h: *n* = 3–5). #*P* < 0.05. The data used to make this figure can be found in S1 Data. DC, dark control; MW, middle-wavelength; OPN5, neuropsin; SW, short-wavelength.
(TIF)

S4 Fig Spectral sensitivity of mouse retinal photoreceptors and spectrum of LED light. A. Normalized sensitivity of photoreceptors based on Govardovkii’s nomograms [42] and adapted to melanopsin and OPN5 (based on [37,51]). B. Summary of the normalized sensitivity of the photopigments at each wavelength used in the present study. C. Peaks and half-bandwidth of the LEDs used in this study. All values are normalized (purple LED, λ_max_ = 395 nm, half-bandwidth = 8 nm; blue LED, λ_max_ = 465 nm, half-bandwidth = 15 nm; λ_max_ = 520 nm, half-bandwidth = 16 nm). The data used to make this figure can be found in S1 Data. LED, light-emitting diode; OPN5, neuropsin.
(TIF)

S5 Fig A. Mean light-induced phase shift in heterozygous genotypes. B. Difference in the endogenous period before and after the light stimulation in heterozygous genotypes. A positive value corresponds to a lengthening of the period. Bars represent mean ± SEM (DC: *n* = 17; WT: *n* = 5–6 for heterozygous photoreceptor-deficient mice:). C. Effect of the absence of one type of photoreceptor on the endogenous period of the retinal clock. The endogenous period is calculated on a 3-day baseline before light stimulation in retinal explants from *Per2*^*Luc*^ mice and photoreceptor-deficient mice. Bars represent mean ± SEM (WT: *n* = 8; for homozygous photoreceptor-deficient mice: *n* = 5–6; for heterozygous photoreceptor-deficient mice: *n* = 3–11). Statistical differences with the WT are indicated by **P < 0.001. The data used to make this figure can be found in S1 Data. DC, dark control; WT, wild-type.
(TIF)

S6 Fig Relative expression of cone opsins, melanopsin, OPN5, and *Per2* in the retina of WT and rodless (*Nrl*^−/−^) mice. A. Photomicrographs of retinal sections from 4-week-old WT and *Nrl*^−/−^ mice counterstained with cresyl violet. *Nrl*^−/−^ retina appears grossly normal at this age with sparse rosette-like structures indicating abnormal organization of photoreceptors (white arrow). Scale = 50 μm. B. Relative opsins (SW, MW, rhodopsin), melanopsin, and OPN5 mRNA levels in the retina of WT (black bars) and *Nrl*^−/−^ (grey bars) mice determined by using real-time RT-PCR. Results are expressed as mean ± SEM (*n* = 6 for each genotype). The *Nrl*^−/−^ knockout mouse is characterized by a total absence of rhodopsin and overexpression of SW and MW opsins. The relative quantity of melanopsin is also up-regulated, whereas OPN5 levels are equivalent in both genotypes. The level of *Per2* is not altered in the *Nrl*^−/−^ mice. **P < 0.01. The data used to make this figure can be found in S1 Data. GCL, ganglion cell layer; INL, inner nuclear layer; MW, middle-wavelength; *Nrl*, retina-specific leucine zipper protein; ONL, outer nuclear layer; OPN5, neuropsin; *Per2*, *Period 2*; RT-PCR, reverse transcription PCR; SW, short-wavelength; WT, wild-type.
(TIF)

## Abbreviations

CBX: carbenoxolone
CT: circadian time
DC: dark control
ipRGC: intrinsically photosensitive melanopsin-containing retinal ganglion cell
LED: light-emitting diode
Luc: Luciferase
MW: middle-wavelength
NIF: non-image forming
*Nrl*: retina-specific leucine zipper protein
*Opn4*: melanopsin
OPN5: neuropsin
PER2: PERIOD 2
PER2::Luc: PERIOD2::Luciferase
RPE: retinal pigment epithelium
RT-PCR: reverse transcription PCR
SCN: suprachiasmatic nucleus
SW: short-wavelength
*TRβ*: thyroid hormone receptor beta
WT: wild-type
ZT: zeitgeber time
