# Supplementary figures and images for: Rods contribute to the light-induced phase shift of the retinal clock in mammals
Source: PLoS Biol. 2019 Mar 1;17(3):e2006211. doi: 10.1371/journal.pbio.2006211 (PMC6415865; doi:10.1371/journal.pbio.2006211)

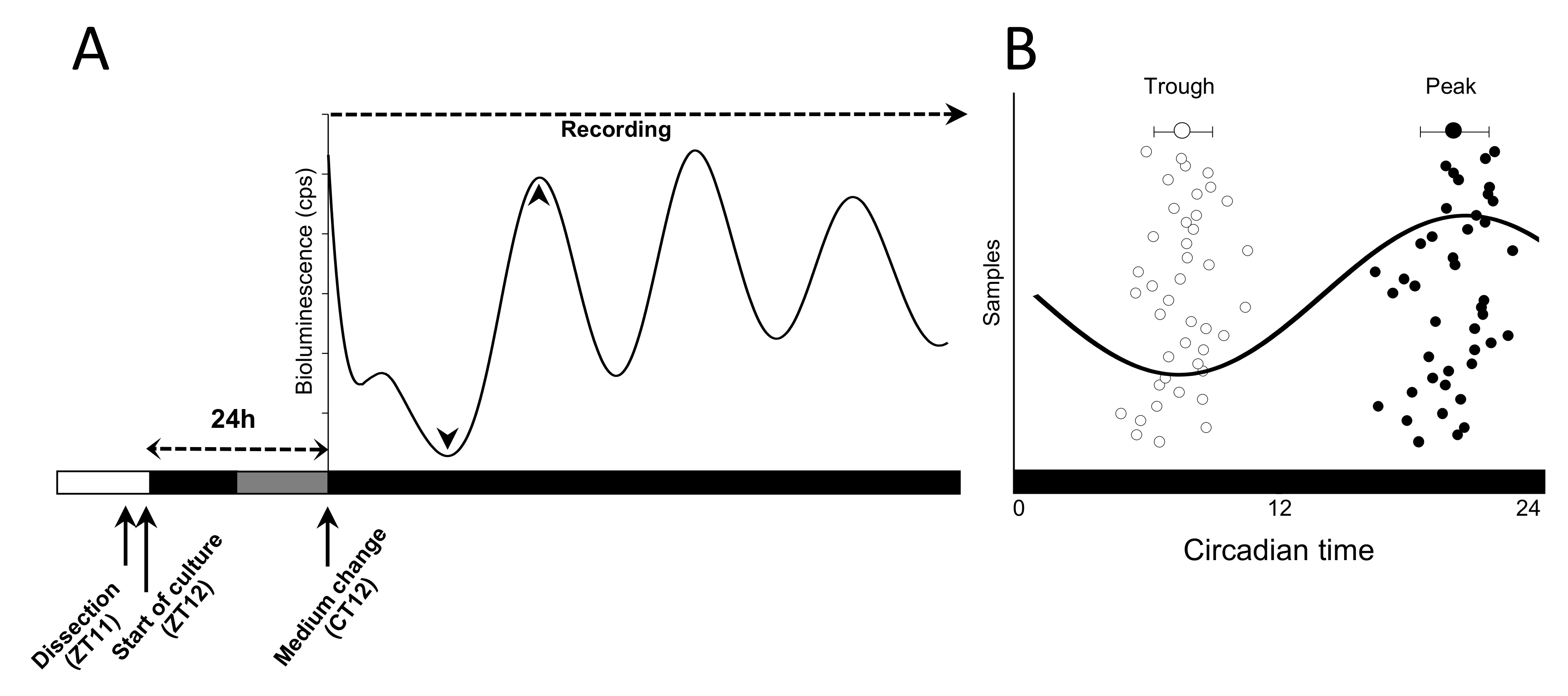

Supplement: S1 Fig — A. Schematic representation of the protocol used for the retinal explant culture and calculation of the circadian time of the oscillation. Retinal explants were dissected at ZT11 and cultured just before light offset (ZT12). The projected ZT12 is then considered as CT12 and used to predict the circadian time of the retinal clock in vitro. Arrowheads correspond to the trough and the peak of the first complete oscillation. B. The first trough (white circles) and peak (black circles) of PER2::Luc oscillation occur, respectively, at CT 7.65 ± 1.33 and CT 19.94 ± 1.55 (mean ± SD). Each circle on the same line represents the trough and the peak of the same retinal explant (n = 42). The data used to make this figure can be found in S1 Data. CT, circadian time; PER2::Luc, PERIOD2::Luciferase; ZT, zeitgeber time. (TIF) [file pbio.2006211.s002.tif]

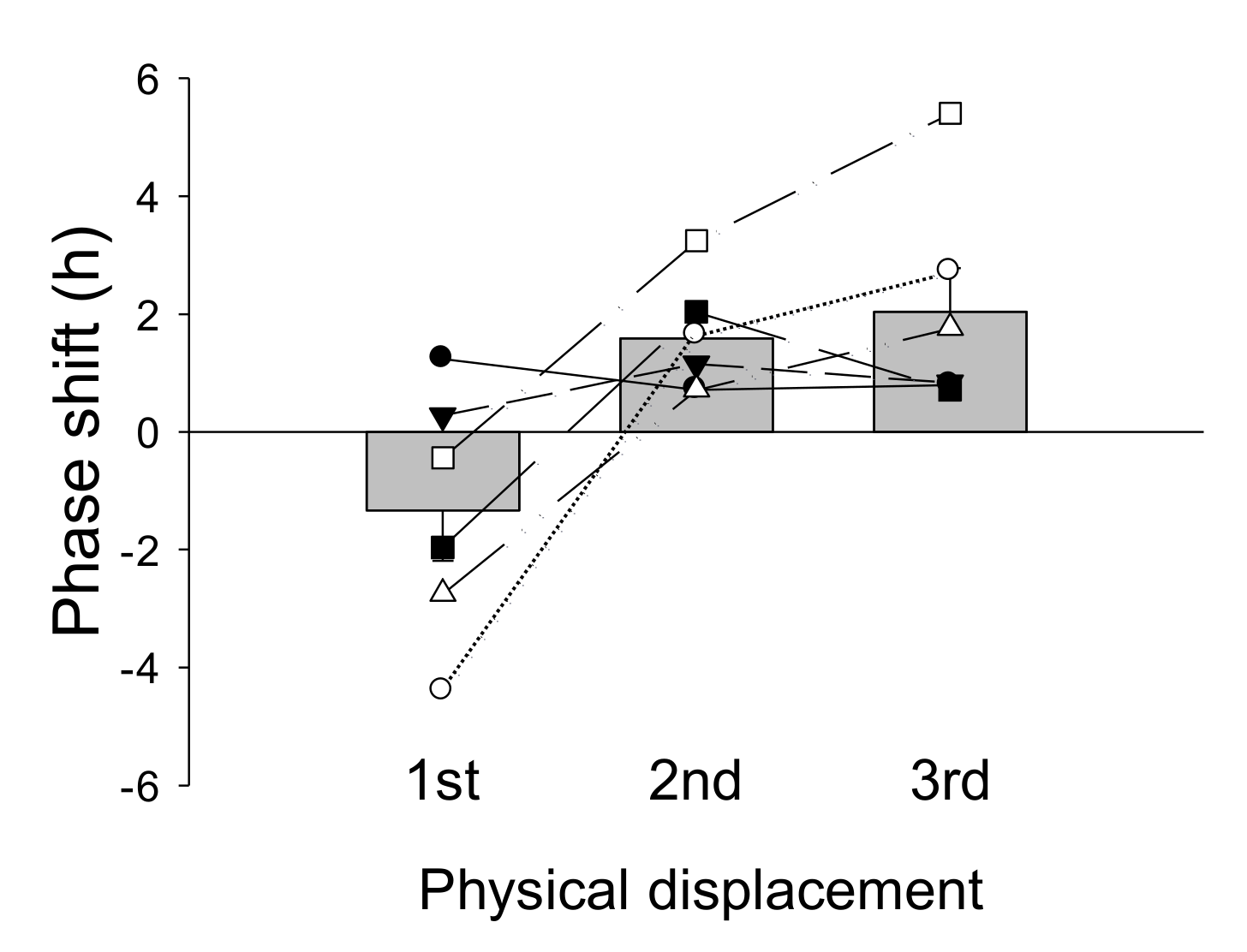

Supplement: S2 Fig — For light-induced phase shift experiments of the retinal clock, the classical procedure involves the transfer of the cultured tissue into a light stimulator outside the Lumicycle. The effect of physical displacement on the phase of PER2::Luc expression was analyzed following three successive displacements of the culture dishes. We show for the same retinal explant a robust and random effect of displacement on the phase of PER2::Luc (advance or delay) that may simply result from a medium homogenization. Each symbol corresponds to an individual explant (n = 6). Bars represent the mean ± SEM. The data used to make this figure can be found in S1 Data. PER2::Luc, PERIOD2::Luciferase. (TIF) [file pbio.2006211.s003.tif]

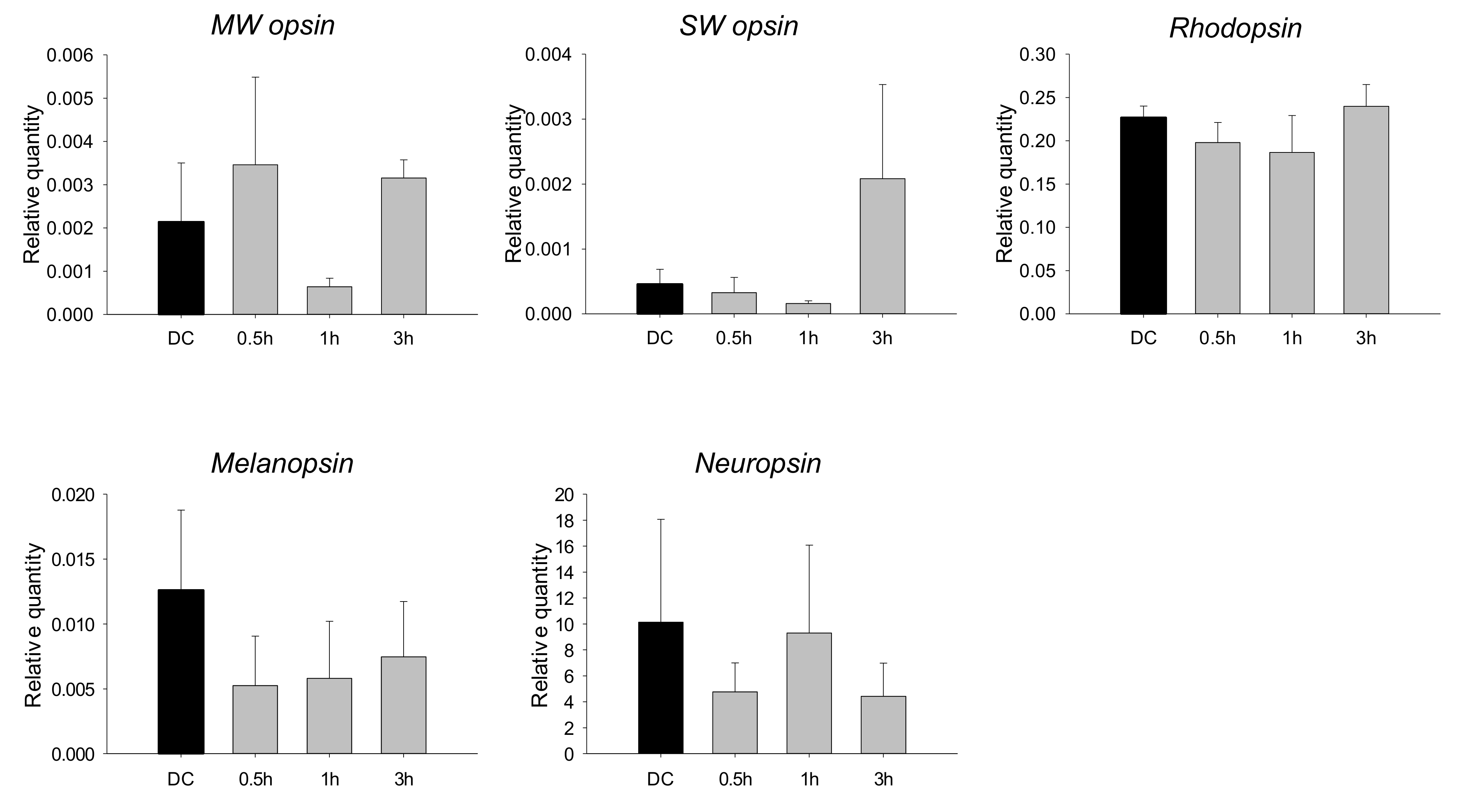

Supplement: S3 Fig — Relative expression of opsins (MW opsin, SW opsin, rhodopsin, melanopsin, and OPN5) of 10-day-cultured retinas stimulated by different durations (0.5 h, 1 h, and 3 h; grey bars) at 465 nm was compared to DC retinas (black bars). Bars represent mean ± SEM (DC: n = 3; 0.5–3 h: n = 3–5). #P < 0.05. The data used to make this figure can be found in S1 Data. DC, dark control; MW, middle-wavelength; OPN5, neuropsin; SW, short-wavelength. (TIF) [file pbio.2006211.s004.tif]

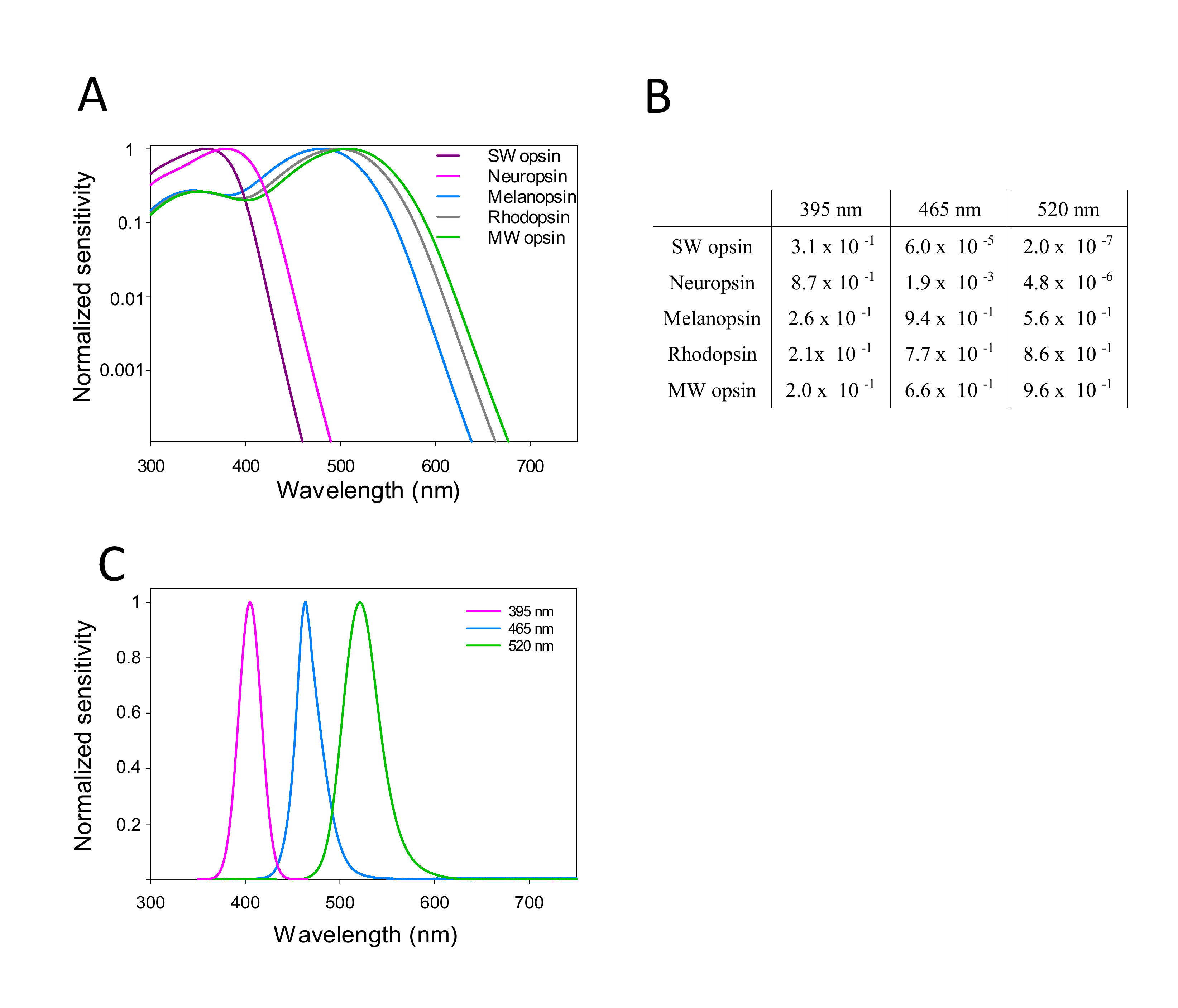

Supplement: S4 Fig — A. Normalized sensitivity of photoreceptors based on Govardovkii’s nomograms [42] and adapted to melanopsin and OPN5 (based on [37,51]). B. Summary of the normalized sensitivity of the photopigments at each wavelength used in the present study. C. Peaks and half-bandwidth of the LEDs used in this study. All values are normalized (purple LED, λmax = 395 nm, half-bandwidth = 8 nm; blue LED, λmax = 465 nm, half-bandwidth = 15 nm; λmax = 520 nm, half-bandwidth = 16 nm). The data used to make this figure can be found in S1 Data. LED, light-emitting diode; OPN5, neuropsin. (TIF) [file pbio.2006211.s005.tif]

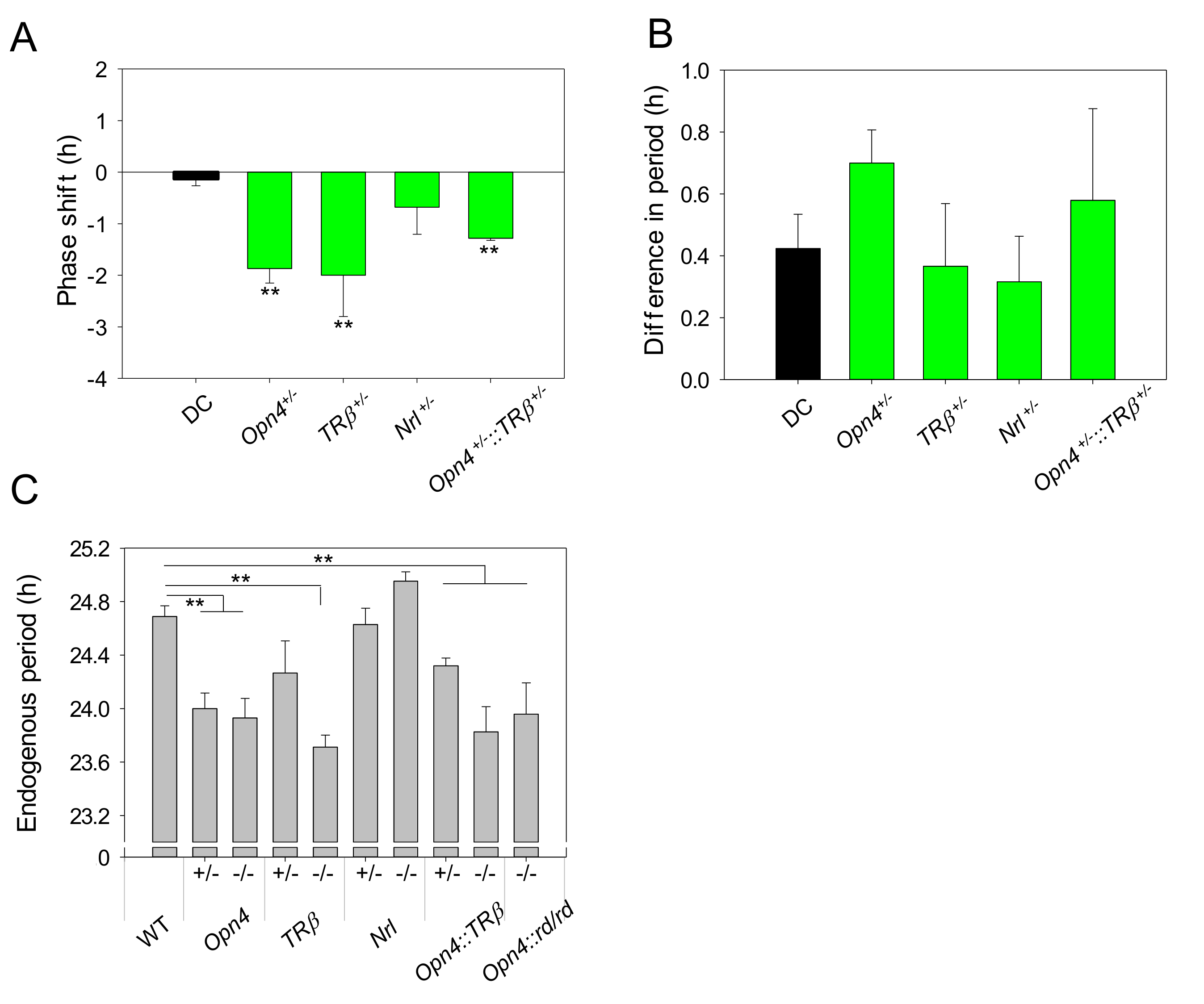

Supplement: S5 Fig — A. Mean light-induced phase shift in heterozygous genotypes. B. Difference in the endogenous period before and after the light stimulation in heterozygous genotypes. A positive value corresponds to a lengthening of the period. Bars represent mean ± SEM (DC: n = 17; WT: n = 5–6 for heterozygous photoreceptor-deficient mice:). C. Effect of the absence of one type of photoreceptor on the endogenous period of the retinal clock. The endogenous period is calculated on a 3-day baseline before light stimulation in retinal explants from Per2Luc mice and photoreceptor-deficient mice. Bars represent mean ± SEM (WT: n = 8; for homozygous photoreceptor-deficient mice: n = 5–6; for heterozygous photoreceptor-deficient mice: n = 3–11). Statistical differences with the WT are indicated by **P < 0.001. The data used to make this figure can be found in S1 Data. DC, dark control; WT, wild-type. (TIF) [file pbio.2006211.s006.tif]

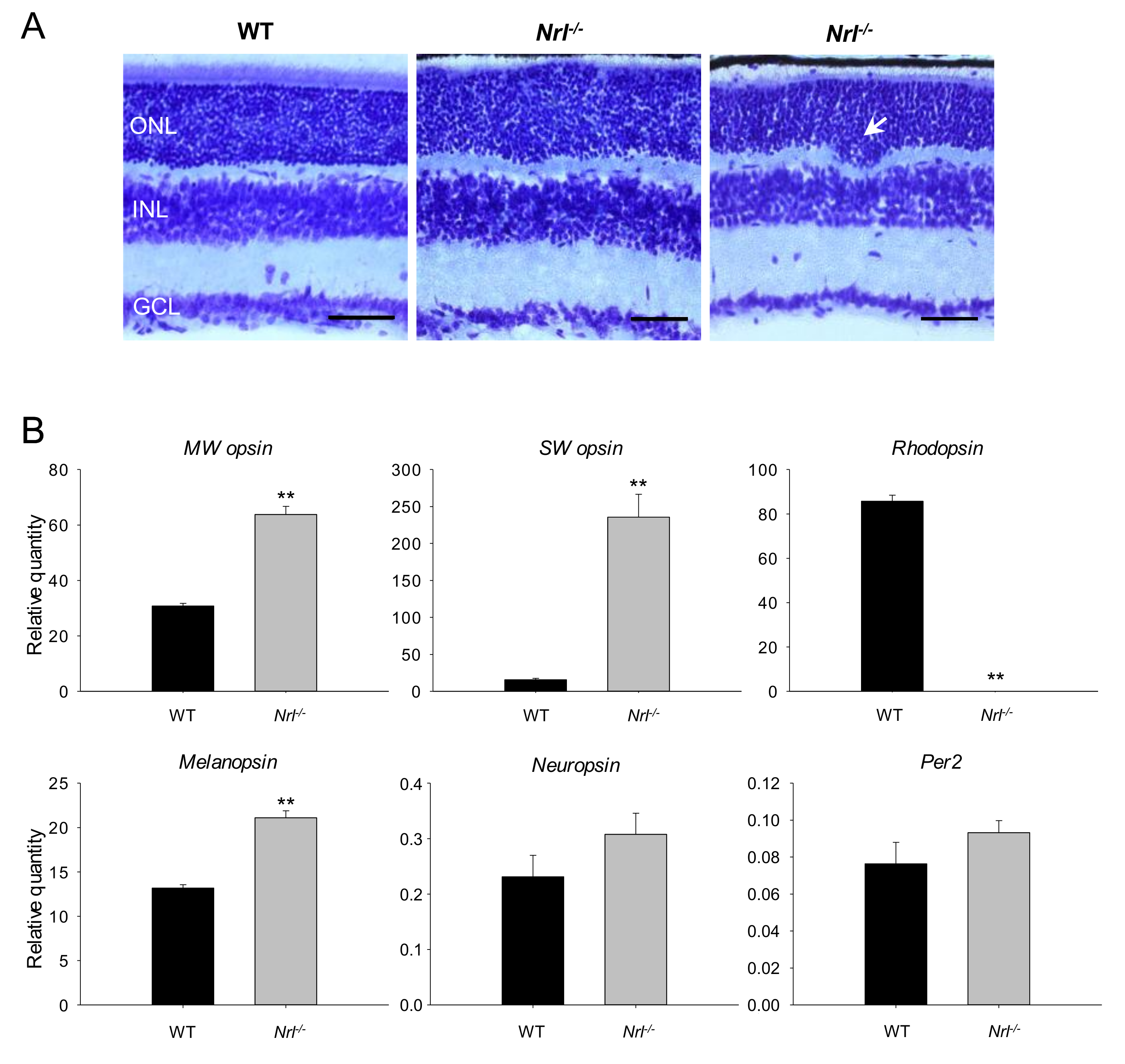

Supplement: S6 Fig — A. Photomicrographs of retinal sections from 4-week-old WT and Nrl−/− mice counterstained with cresyl violet. Nrl−/− retina appears grossly normal at this age with sparse rosette-like structures indicating abnormal organization of photoreceptors (white arrow). Scale = 50 μm. B. Relative opsins (SW, MW, rhodopsin), melanopsin, and OPN5 mRNA levels in the retina of WT (black bars) and Nrl−/− (grey bars) mice determined by using real-time RT-PCR. Results are expressed as mean ± SEM (n = 6 for each genotype). The Nrl−/− knockout mouse is characterized by a total absence of rhodopsin and overexpression of SW and MW opsins. The relative quantity of melanopsin is also up-regulated, whereas OPN5 levels are equivalent in both genotypes. The level of Per2 is not altered in the Nrl−/− mice. **P < 0.01. The data used to make this figure can be found in S1 Data. GCL, ganglion cell layer; INL, inner nuclear layer; MW, middle-wavelength; Nrl, retina-specific leucine zipper protein; ONL, outer nuclear layer; OPN5, neuropsin; Per2, Period 2; RT-PCR, reverse transcription PCR; SW, short-wavelength; WT, wild-type. (TIF) [file pbio.2006211.s007.tif]
